# Primary extranodal marginal zone lymphoma of gastric mucosa-associated lymphoid tissue in children: a case report and review of the literature

**DOI:** 10.1186/s13256-026-06405-0

**Published:** 2026-07-24

**Authors:** Ying Yang, Lei Lei, Haiyan Gao, Jing Liao

**Affiliations:** 1 Department of Pediatrics, School of Medicine, Sichuan Provincial People’s Hospital, University of Electronic Science and Technology of China, No.32 West Second Section First Ring Road, Chengdu, 610072 Sichuan Province China; 2 Department of Gastroenterology and Hepatology, School of Medicine, Sichuan Provincial People’s Hospital, University of Electronic Science and Technology of China, No.32 West Second Section First Ring Road, Chengdu, 610072 Sichuan Province China; 3 Department of Nuclear Medicine, School of Medicine, Sichuan Provincial People’s Hospital, University of Electronic Science and Technology of China, No.32 West Second Section First Ring Road, Chengdu, 610072 Sichuan Province China

**Keywords:** Gastric mucosa-associated lymphoid tissue lymphoma, Pediatric, *Helicobacter pylori* infection, Antigen-driven, Case report

## Abstract

**Background:**

Extranodal marginal zone lymphoma of mucosa-associated lymphoid tissue represents an important subtype of marginal zone lymphoma, a heterogeneous B-cell neoplasm originating from the marginal zone of lymphoid follicles. Therefore, gastric marginal zone lymphoma arising from mucosa-associated lymphoid tissue in immunocompetent children is particularly special. This article aims to report this rare case and systematically review the research progress of pediatric marginal zone lymphoma combined with its clinicopathological features.

**Case presentation:**

A 13-year-old Chinese female presented with a 3-month history of recurrent epigastric pain. Gastroscopy revealed multiple ulcers in the gastric angle and antrum. Histopathological examination identified lymphoepithelial lesions and lymphoid infiltration. Immunohistochemistry showed positivity for CD20 and CD79a and negativity for CD3. PCR and capillary electrophoresis detected clonal immunoglobulin gene rearrangements, confirming monoclonality. These findings supported the diagnosis of gastric mucosa‑associated lymphoid tissue lymphoma. [^68^ Ga]Pentixafor-PET showed multiregional involvement, and Lugano stage IV was suspected (based on imaging alone, as histopathological confirmation was declined by the family). Fortunately, the patient's gastric lesions are now resolved without any discomfort after *Helicobacter pylori* eradication therapy. Nearly 12 months later, the patient remains on follow-up and is currently asymptomatic.

**Conclusions:**

This case describes a Chinese child with pediatric gastric mucosa‑associated lymphoid tissue lymphoma with PET‑defined stage IV disease that was clearly associated with *Helicobacter pylori* infection. The patient achieved clinical and endoscopic remission with eradication therapy. These findings suggest that even when imaging indicates possible distant involvement, some children may still benefit from conservative treatment targeting the infection source, thereby avoiding more aggressive initial therapy (such as chemotherapy) and expanding clinical management options for such rare cases.

## Background

Marginal zone lymphomas (MZL) represent a group of B-cell neoplasms originating from the marginal zone of secondary lymphoid follicles, demonstrating significant clinical and biological heterogeneity. Accounting for approximately 7.8% of all non-Hodgkin lymphoma (NHL). The disease may involve lymph nodes, spleen, and various extranodal mucosal lymphoid tissues [1]. Among MZL subtypes, extranodal marginal zone lymphoma of mucosa-associated lymphoid tissue (MALT lymphoma) is the most common, exhibiting a strong predilection for the stomach [2].

The pathogenesis of MALT lymphoma is linked to chronic antigen stimulation. Most cases originate from mucosal sites associated with persistent infection or autoimmune diseases. For instance, gastric MALT lymphoma is predominantly associated with *Helicobacter pylori* (*H. pylori*) infection. At the same time, cases involving the ocular adnexa, lung, and salivary glands are linked to Chlamydia psittaci, hepatitis C virus, and autoimmune conditions such as Sjögren's syndrome and Hashimoto's thyroiditis, respectively [3–9]. Of note, MALT lymphoma is exceptionally rare in the pediatric age group, with only sporadic case reports documented in the literature, some of which have been associated with HIV infection [10].

From a broader disease perspective, the concept of primary gastrointestinal lymphoma was first introduced in the 1960s. Primary gastrointestinal lymphoma accounts for 10–20% of all NHL cases and represents 40–50% of all extranodal lymphomas [11, 12]. In pediatric primary gastrointestinal lymphoma, the intestinal tract is the most frequently involved site (approximately 66%), whereas primary gastric lymphoma accounts for only about 10% of cases [13, 14]. According to 41 years of data (1973–2014) from the US SEER (Surveillance, Epidemiology, and End Results) database, among 452 pediatric cases of primary gastric lymphoma, the gastric antrum was the most common tumor location (46%). The vast majority of cases (88.5%) exhibited aggressive B-cell phenotypes, such as Burkitt lymphoma (34.6%) and diffuse large B-cell lymphoma (23%) [14]. Within this context, the occurrence of gastric MALT lymphoma in a pediatric patient is particularly notable. This article presents a case of gastric MALT lymphoma in an immunocompetent Chinese child. It is unique because that is a very uncommon presentation, especially with multiregional involvement at diagnosis (suspected Lugano stage IV based on PET imaging alone). Although most pediatric gastric lymphomas are aggressive B-cell types, this case demonstrates which is a very uncommon presentation can achieve clinical and endoscopic remission with eradication of *H. pylori*, without the need for chemotherapy or other invasive interventions.

This provides real-world evidence to the scientific literature that a conservative, antibiotic-first approach may be effective even for nonlocalized pediatric MALT lymphomas, thus providing support for a de-escalation approach to specific cases. It also highlights the importance of considering MALT lymphoma in the differential diagnosis of upper gastrointestinal symptoms in children and highlights the challenges and potential alternatives to aggressive staging in children with limited family consent.

## Case presentation

**Fig. 1:**
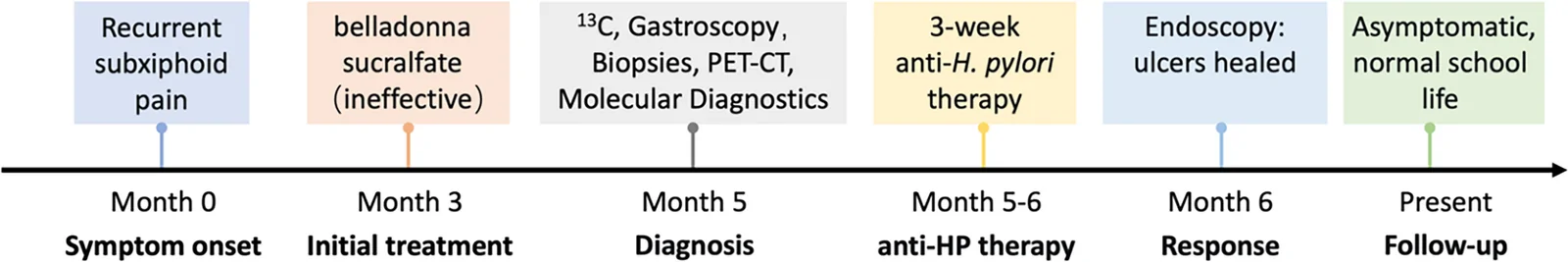
Patient timeline of clinical presentation, diagnosis, and management

A 13-year-old Chinese female patient presented to our pediatric outpatient, with a 3-month history of recurrent epigastric pain. The patient lives with her parents and attends school regularly, with no significant psychosocial stressors identified. No significant psychosocial risk factors were identified. On abdominal examination, mild subxiphoid tenderness was elicited, with no muscle tension, rebound tenderness, or palpable masses. The patient initially declined gastroscopic examination and received symptomatic medication, including sucralfate, belladonna mixture, and lactobacillus, which provided no significant clinical improvement. 5 months later, the patient returned for follow-up. *H. pylori* infection was confirmed by ^13^C-urea breath test and anti-*H. pylori* therapy was started. The time sequence of clinical events in this child is shown in Fig. 1.

**Fig. 2:**
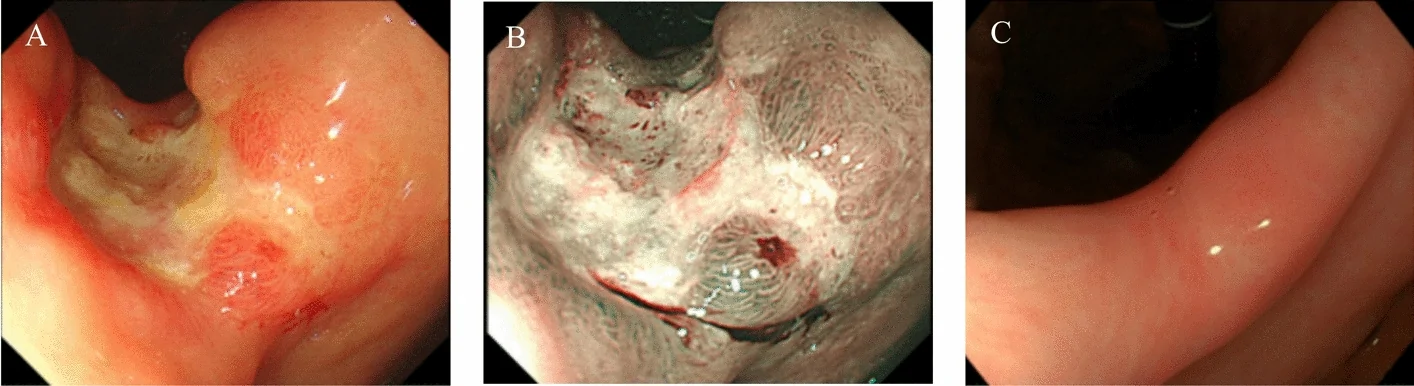
Gastroscopic images. **A** There was an irregular ulcer of the stomach, covered with white fur, and the surrounding mucosa was hyperemic and edematous. **B** Narrow-band imaging showing distortion of the microvascular architecture in the gastric mucosa surrounding the ulcerative lesions. **C** The ulcer improved after anti-*Helicobacter pylori* treatment

**Fig. 3:**
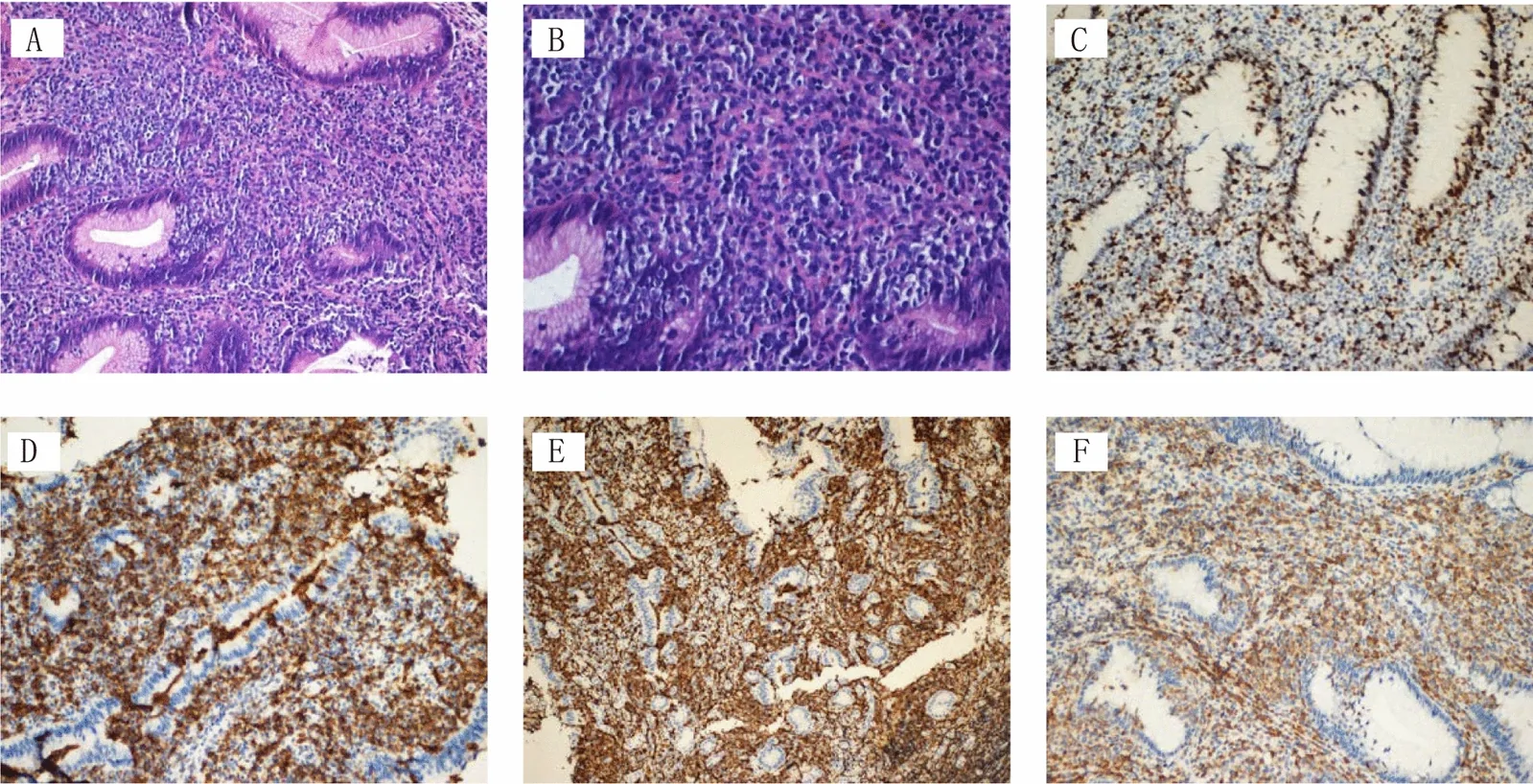
Histopathological and immunohistochemical features of gastric mucosa-associated lymphoid tissue lymphoma. **A**, **B** Hematoxylin and eosin staining of the gastric antrum/angle tissue. **A** Low-power view (× 10) and **B** high-power view (× 20) demonstrate dense, deeply stained lymphocytic infiltration within the epithelial layer. Characteristic lymphoepithelial lesions are present, with scattered or clustered lymphoma cells infiltrating and destroying the glandular epithelium. **C**–**F** Immunohistochemical staining of the neoplastic cells. The tumor cells show positive expression for **C** cluster of differentiation 20 (pan-B-cell marker), **D** cluster of differentiation 79a (B-cell lineage marker), and **F** B-cell leukemia/lymphoma 2 (approximately 50%), **E** Ki-67 proliferation index is low (approximately 15%), consistent with the indolent nature of mucosa-associated lymphoid tissue lymphoma

Gastroscopy revealed an irregular, approximately 3 cm diameter ulcer in the gastric angle, covered with white exudate and surrounded by congested and edematous mucosa. Multiple similar ulcers (0.3–2.0 cm in diameter) were observed in the gastric antrum (Fig. 2). Histopathological examination of the gastric biopsy specimens demonstrated prominent lymphoid proliferation within the gastric mucosal stroma, notably with characteristic lymphoepithelial lesions. Immunophenotypic analysis demonstrated a CD20 + /CD79a + /CD3- profile, consistent with a B-cell lineage. Bcl-2 positivity aligned with the phenotypic characteristics of MALT lymphoma The low Ki-67 proliferation index (approximately 15%) indicated an indolent biological behavior (Fig. 3). Furthermore, clonal rearrangements of the IgH and IgK genes were detected by PCR and capillary electrophoresis, providing definitive molecular evidence of monoclonality and confirming a neoplastic B‑cell proliferation consistent with MALT lymphoma.

**Fig. 4:**
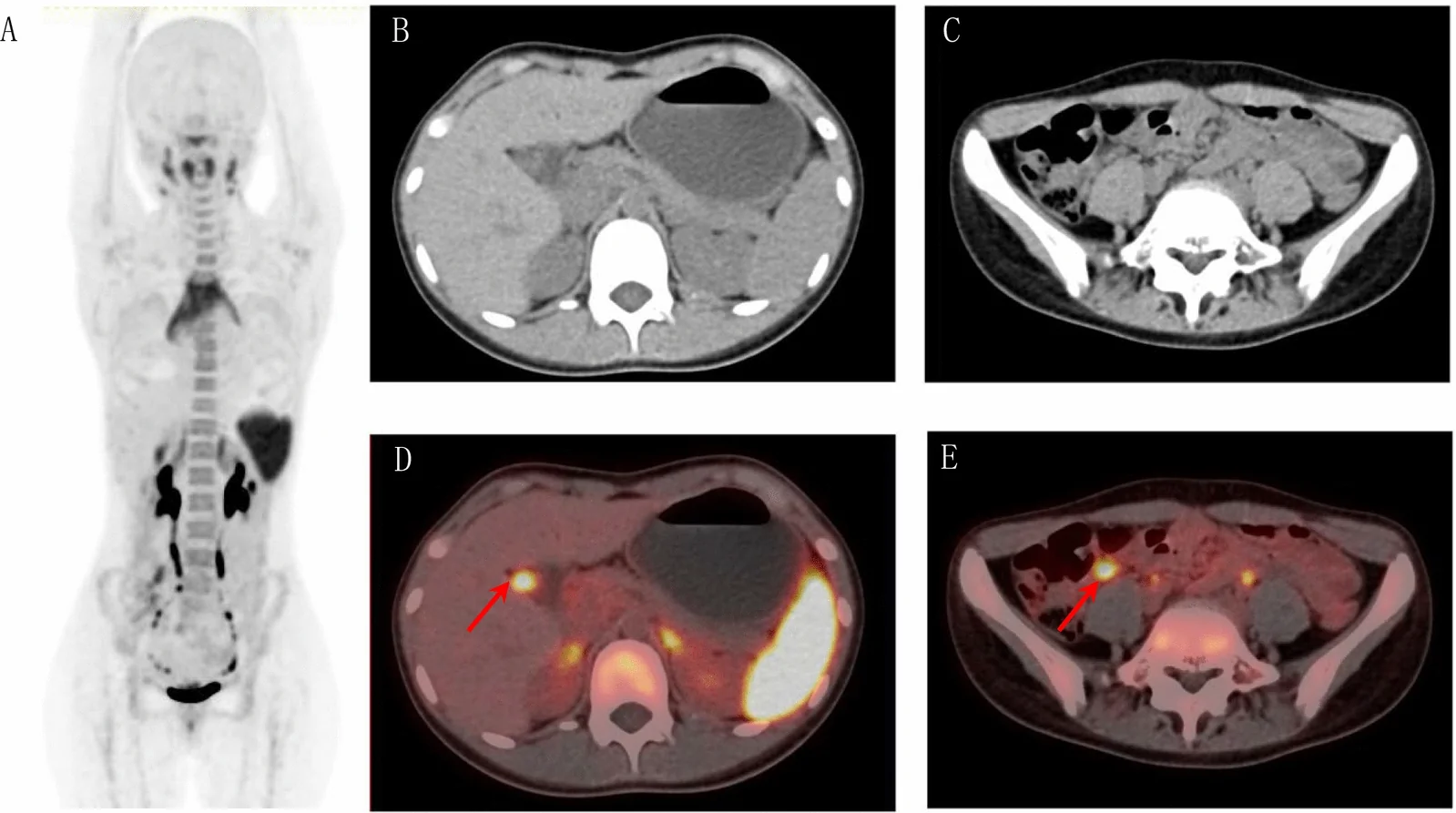
[⁶⁸Ga]Pentixafor-positron emission tomography/computed tomography imaging findings in gastric mucosa-associated lymphoid tissue lymphoma. **A** Whole-body [⁶⁸Ga]Pentixafor-positron emission tomography maximum intensity projection image. **B**, **C** Axial computed tomography images at the level of the gastric antrum (**B**) and the ileocecal region (**C**) demonstrating mild wall thickening and an enlarged mesenteric lymph node, respectively. **D**, **E** Corresponding axial [⁶⁸Ga]Pentixafor-positron emission tomography images show focal increased tracer uptake in the gastric antrum (**D**, arrow) and in the right ileocecal mesenteric lymph node (**E**, arrow), consistent with C-X-C chemokine receptor type 4-expressing lymphoma involvement. The positron emission tomography-avid mesenteric node was not histopathologically confirmed due to family refusal of biopsy

Moreover, [^68^ Ga]Pentixafor-PET imaging demonstrated: (1) mildly increased CXCR4 receptor expression in the gastric, suggestive of lymphoma; (2) enlargement of mesenteric lymph nodes in the right ileocecal region with elevated CXCR4 expression (SUVmax 8.46, delayed imaging SUVmax 8.70), indicating probable lymphomatous involvement (Fig. 4). These findings were consistent with suspected Lugano stage IV disease (Table 1) [15].

**Table 1:** Lugano staging system for gastrointestinal tract lymphoma [15]

Stage			Extent of invasion
Stage I	Tumor confined to the GI tract		Mucosa, submucosa, muscularis propria, serosal layer
Stage II	Tumor extending into the abdomen from the primary GI site	Stage II_1_	The perigastric lymph nodes were involved
		Stage II_2_	More distal regional lymph nodes were involved
Stage II_E_	Penetrates the serosal layer and involves adjacent organs or tissues, with/without abdominal lymph node metastasis		
Stage IV	Disseminated extranodal involvement or associated with supraphrenic lymph node involvement		

GI: Gastrointestinal

Following a multidisciplinary team discussion, the final diagnosis was PET-defined stage IV disease of MALT lymphoma. The patient received amoxicillin (50 mg/kg/day, divided into two doses), metronidazole (20 mg/kg/day, divided into two doses), omeprazole (1 mg/kg/day) for 14 days, combined with sucralfate (as per pediatric dosing). Although sucralfate is not a standard component of *H. pylori* eradication therapy, it was added for its mucosal protective effects given the presence of multiple gastric ulcers at diagnosis. It has no known direct anti-lymphoma activity, and the complete histological remission achieved is unequivocally attributable to *H. pylori* eradication rather than to sucralfate. Her abdominal pain resolved within 3 weeks, and a follow-up ^13^C-urea breath test 1 month later was negative. The patient did not report any significant side effects (such as severe nausea, diarrhea, or allergic reactions). At the first follow-up, the patient remained asymptomatic, and repeat gastroscopy showed marked improvement of the gastric ulcerative lesions. She is currently under planned long-term surveillance. Subsequent gastroscopy showed scar formation at the previous lesion site, and MRI indicated localized wall th ickening in the gastric angle. The patient is grateful to have avoided more invasive therapies and to have returned to a normal life. A key limitation is the family's refusal of colonoscopy and lymph node biopsy, which introduces uncertainty in staging. Without histopathological confirmation, we cannot definitively exclude concurrent gastrointestinal involvement or confirm the nature of PET-avid nodal disease. This could theoretically lead to either under-staging (missed lesions) or over-staging (reactive nodes). This case demonstrates that even PET-defined advanced-stage disease, suspected to be Lugano stage IV based on imaging alone, may achieve clinical and endoscopic remission with *H. pylori* eradication alone.

## Discussion

This case documents a rare pediatric gastric MALT lymphoma in an immunocompetent adolescent who achieved clinical and endoscopic remission with *H. pylori* eradication alone, despite PET findings suggestive of advanced-stage (Lugano IV) disease, which predominantly affects individuals over 60 years of age [16, 17].

The rarity of gastric MALT lymphoma in children compared to its relative frequency in adults is multifactorial. A key factor lies in differences in immune response: children predominantly mount a Treg-mediated tolerogenic immune response to *H. pylori* infection, resulting in significantly lower gastric mucosal inflammation than in adults, thereby reducing the driving force for lymphoid proliferation and malignant transformation [18, 19]. In addition, the development of MALT lymphoma typically requires decades of chronic antigenic stimulation, even though *H. pylori* infection is usually acquired in childhood [18]. Furthermore, the accumulation of genetic alterations—critical abnormalities such as t(11;18) require time to develop and accumulate, and this process is usually not completed during childhood [19]. Against this background, the uniqueness of this case lies precisely in the fact that, despite the patient's immature immune system, the complete process from infection to malignant transformation was already completed in childhood, suggesting the possible presence of host genetic susceptibility or bacterial virulence factors that may accelerate disease progression.

Some studies have shown that compared with CT, CXCR4-directed PET/CT may improve the current diagnostic algorithm and affect the management relative to CT alone, potentially avoiding some biopsies [20]. In newly diagnosed marginal zone lymphomas (MZL) patients, compared with [18F]FDG, [^68^ Ga]Pentixafor identified more disease sites, and chemokine receptor-targeted imaging improved diagnostic readout regardless of the MZL subtype [21] Using this approach, [⁶⁸Ga]Pentixafor-PET in our case revealed CXCR4-avid lesions not only in the stomach but also in multiple lymph node groups, including the ileocecal region, consistent with Lugano stage IV disease. We explicitly acknowledge that this use for initial staging is investigational, not standard-of-care and this staging is presumptive, as no tissue biopsy was obtained from the mesenteric lymph nodes.

Several limitations must be acknowledged. The most significant is that the staging of this patient as Lugano stage IV is presumptive. The family declined colonoscopy and lymph node biopsy; consequently, the PET‑avid mesenteric lymph nodes were never histopathologically confirmed as lymphomatous involvement, and reactive lymphadenopathy due to chronic *H. pylori* infection cannot be excluded as an alternative explanation for PET avidity.

A second limitation is the 5‑month interval between symptom onset and treatment initiation, which resulted from the family’s initial refusal of gastroscopy. Given the indolent nature of MALT lymphoma, this diagnostic delay is unlikely to have affected the outcome, as the patient achieved remission (clinical and endoscopic). Nevertheless, we emphasize the importance of timely endoscopic evaluation in children with persistent upper gastrointestinal symptoms.

Third, we did not evaluate t(11;18) or other chromosomal translocations in this case, as the family declined further testing. However, the remission achieved with *H. pylori* eradication strongly suggests the absence of t(11;18), a translocation typically associated with antigen independence and antibiotic resistance. Nonetheless, we acknowledge that molecular characterization would strengthen future prospective studies of pediatric MALT lymphoma.

Finally, as a single case report, our findings may not be generalizable to all pediatric patients with gastric MALT lymphoma. Moreover, longer follow‑up is needed to confirm durable remission, given the indolent nature of this disease.

**Fig. 5:**
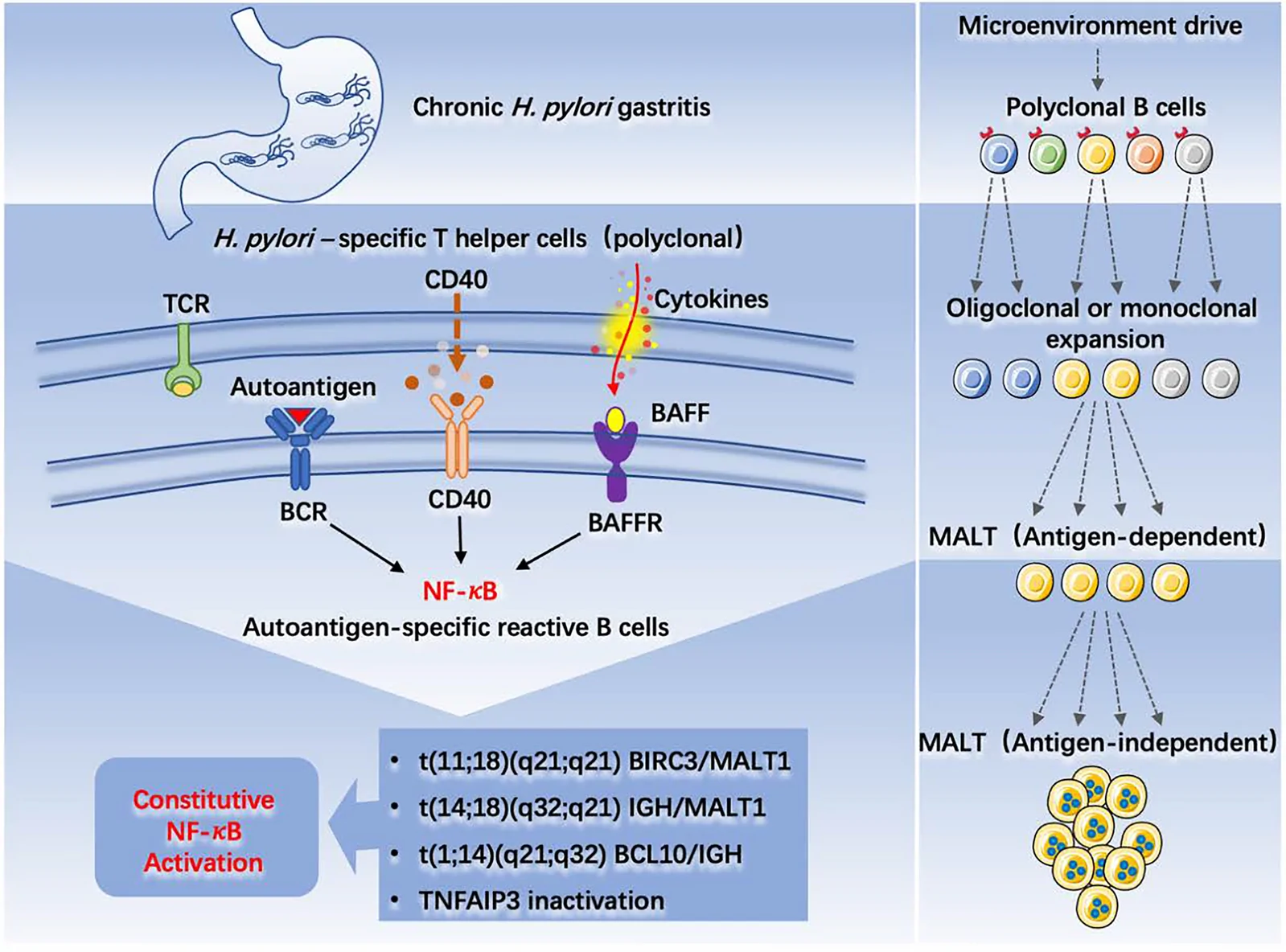
Mechanistic features of gastric mucosa-associated lymphoid tissue lymphoma [25]

The development and progression of gastric MALT lymphoma are closely associated with chronic antigenic stimulation within the local microenvironment, although the precise target antigens involved remain largely unidentified [22]. Notably, this antigen-driven process may not originate from direct recognition of pathogens (such as *H. pylori*), but is more likely to represent a concomitant, indirect immune response against self-antigens [23, 24]. This process serves as a classic model for studying antigen-driven malignant transformation of B cells: In the context of *H. pylori* infection, activated specific helper T cells provide cytokine and co-stimulatory signals (for example, CD40L and BAFF), thereby supporting polyclonal B cells that recognize cross-reactive self-antigens in the gastric mucosa via their B-cell receptors. This sustained T-cell-dependent stimulation ultimately drives the selection and expansion of antigen-dependent MALT lymphoma clones (Fig. 5) [25]. In summary, the pathogenesis of gastric MALT lymphoma is a multi-step process.

Therapeutic decision-making for gastric MALT lymphoma is highly dependent on disease stage and *H. pylori* infection status, with the core objectives being disease control, maximal preservation of gastric function, and minimization of treatment-related morbidity [26]. Successful eradication can lead to both clinical and histological complete remission in localized disease [27, 28], and it is recommended that all patients, regardless of disease stage, receive standard *H. pylori* eradication therapy [29]. Following therapy, *H. pylori* eradication was confirmed by a negative urea breath test [30, 31]. In most patients, this approach achieves lymphoma regression and long-term disease control. For patients who fail to respond, stage‑guided treatment options include radiotherapy or systemic therapy [32, 33].

Based on the experience with this case and current literature, we suggest the following monitoring approach for similar pediatric cases managed with *H. pylori* eradication alone: short-term endoscopic follow-up at 3–6 months to confirm histological remission and *H. pylori* eradication; long-term clinical and endoscopic surveillance schedule; recommendations against routine imaging in asymptomatic patients; patient education on warning symptoms. We believe this addition enhances the clinical utility of our case report and provides practical guidance for clinicians managing similar rare cases.

This case report documents an exceptionally rare entity—pediatric gastric MALT lymphoma with PET-defined suspected stage IV disease that achieved clinical and endoscopic remission with *H. pylori* eradication alone, supporting an antigen-driven pathogenesis and a conservative treatment approach in children. The use of CXCR4-directed [⁶⁸Ga]Pentixafor-PET provided detailed disease mapping; however, given the lack of histopathological confirmation of PET-avid sites and the investigational nature of this modality for initial staging, the true disease extent remains presumptive.

## Conclusions

In summary, gastric MALT lymphoma serves as a paradigm for the "antigen stimulation—chronic inflammation—malignant transformation" sequence in lymphomagenesis. Although this pediatric case presented at a PET-defined stage IV disease, the favorable initial response to anti-*H. pylori* therapy highlights the paramount importance of understanding its underlying biology in guiding clinical practice. Long-term follow-up remains essential for such rare cases, and future studies are warranted to establish standardized treatment protocols.

## Data Availability

Not applicable.

## Abbreviations

MZL: Marginal zone lymphomas
NHL: Non-Hodgkin lymphoma
MALT: Mucosa-associated lymphoid tissue
*H. pylori*: *Helicobacter pylori*
GI: Gastrointestinal
BCR: B-cell receptor
PCR: Polymerase chain reaction
PET: Positron emission tomography
HIV: Human immunodefi ciency virus
SUVmax: Maximum standardized uptake value
MRI: Magnetic resonance imaging
FDG: fluorodeoxyglucose
